# Changes of Dentition State in Leukemic Patients during Chemotherapy

**DOI:** 10.3390/ijerph18158193

**Published:** 2021-08-02

**Authors:** Maja Ptasiewicz, Paweł Maksymiuk, Renata Chałas

**Affiliations:** Department of Oral Medicine, Medical University of Lublin, ul. Chodźki 6, 20-093 Lublin, Poland; maja.ptasiewicz@umlub.pl (M.P.); renata.chalas@umlub.pl (R.C.)

**Keywords:** leukemia, DMFT index, chemotherapy, oral health

## Abstract

A number of systemic diseases including hematological disorders have manifestations in the oral cavity region. These manifestations may often represent early signs of the underlying hematopoietic disease and occur frequently in leukemia. Despite the fact that leukemia has long been known to be associated with oral health deterioration, the available literature on this topic consists mostly of case reports, without data to conclude these. The aim of the study was to assess dentition state in leukemic patients during one cycle of chemotherapy and its correlation with blood parameters. The study included 102 adults treated because of leukemia at the Clinic of Haemato-Oncology and Bone Marrow Transplantation at the university hospital in Lublin, Poland. The sample group consisted of 51 women and 51 men aged 22 to 72 (54.07 ± 10.33) with following diagnoses: Acute myelogenous leukemia (AML)—55 patients (53.92%), Chronic lymphocytic leukemia (CLL)—17 patients (16.67%), Acute lymphoblastic leukemia (ALL)—16 patients (15.69%), Chronic myelogenous leukemia (CML)—10 patients (9.80%), Acute promyelocytic leukemia (APL) —3 patients (2.94%), Chronic hairy cell leukemia (HCL)—1 patient (0.98%). DMFT index was used to assess dentition state. After the cycle of chemotherapy, their dentition state changed in terms of decayed, missing and filled teeth and correlated with hematological parameters. Adult patients with leukemia have high dental treatment needs, and high number of missing teeth; thus, a comprehensive and fast dental treatment is necessary to avoid systemic complications and ensure better quality of life.

## 1. Introduction

A number of systemic diseases including hematological disorders have manifestations in the oral cavity region. These manifestations may often represent early signs of the underlying hematopoietic disease and occur frequently in leukemia. The symptoms may point out the initial evidence of the disease.

Leukemia (Lat. leukemia) is a neoplastic disease originating in the hematopoietic system in which all morphotic elements of the blood are formed. It is characterized by an unlimited and irreversible proliferation of immature hematopoietic or lymphatic cells. The proliferation of these cells can occur both in sites typical for hematopoietic activity, as well as in other tissues and organs. These diseases manifest as quantitative and qualitative changes in leukocytes in the bone marrow, spleen, blood and lymph nodes. The changes occur as a result of neoplastic transformation of stem cells originating from the early stages of hematopoiesis. Clinically, as with other hematopoietic and lymphatic system diseases (bone marrow aplasia, lymphomas and others) hemorrhagic diathesis, granulocytopenia, anemia and impaired immunity may occur. Leukemia is more common in men than in women (3:2), and individual types show the maximum incidence at different ages. General division of leukemias may be presented as follows: acute myelogenous leukemia (AML), acute lymphoblastic leukemia (ALL), chronic myelogenous leukemia (CML) and chronic lymphocytic leukemia (CLL). The classification was modified in the course of the development of knowledge about morphology and features of cancer cells. Nowadays, the classification of hematopoietic neoplasms is based on the 2016 WHO classification [1,2,3,4].

Acute leukemia has two incidence peaks: in adolescence and older age, for chronic leukemia, the median age is about 50 years, while in children it is rare. The standardized death rate due to different types of leukemia is about 7 in 100,000 inhabitants; there are race differences and the level of harmful factors, including environmental factors. The cause of leukemia is usually unknown. The exceptions are acute hyperplasia in people exposed to ionizing radiation and some cytostatics—these leukemias are called therapy related AML (t/AML). Cytostatics that contribute to t/AML are alkylating drugs and topoisomerase II inhibitors. In about 20% of leukemia cases, the disease is detected by chance (peripheral blood counts taken for various reasons). Some patients develop general symptoms. Other symptoms are related to anemia or thrombocytopenia. Hyperviscosity syndrome caused by high leukocytosis may develop. The symptoms of hyperviscosity syndrome are the onset or worsening of heart failure, hypertension, disturbances of consciousness, including coma. These symptoms result from impaired oxygenation of vital organs. There is also a vascular diathesis. Abdominal pain due to enlargement of the liver and/or spleen, bone pain may occur. The most common clinical signs of leukemia are weight loss, fever, anemia, hemorrhage, hepatosplenomegaly and lymphadenopathy. In addition to the lymphatic system, neoplastic cells are located in the bones, bone marrow and central nervous system, giving clinical signs from these tissues and organs [5,6,7,8].

Clinically, leukemia is manifested by oral symptoms, and often the first symptom of the disease is bleeding gums and petechiae within the oral mucosa. Other symptoms may include inflammatory and specific infiltrates, erosions of the oral mucosa, tongue pain and burning, ulcers and necrotic lesions, swelling and gingival hyperplasia. Patients have impaired healing due to reduced anti-inflammatory defense, and chemotherapy can cause clinical changes in the periodontium and mucosa, often difficult to distinguish from the underlying disease. Chemotherapy also contributes to secondary bacterial, viral and fungal infections. After chemotherapy, general symptoms such as permanent cervical lymphadenopathy, malaise, pallor because of anemia or ulcerations related to the immune deficiency may occur. The listed symptoms may be accompanied by gingival bleedings, bleedings from the nose, petechiae, general weakness, malaise and mild fever. Therefore dentists/ oral medicine specialists play an important role in therapy of patients presenting these common symptoms both of the disease itself and its treatment [9,10,11,12,13].

### AIM

Presence of pathological changes in soft tissues of the oral cavity among patients with leukemia is well-documented. The data on the state of these patients’ dentition, which is also important in oral health, is still limited. The present study aimed to assess the dentition state in leukemic patients during one cycle of chemotherapy.

## 2. Materials and Methods

### 2.1. Participant Selection

The study included an accessible sample of leukemic patients being treated with chemotherapy for different types of leukemia in the Clinic of Haemato-Oncology and Bone Marrow Transplantation of the Independent Public Clinical Hospital No. 1 in Lublin, Poland. This hospital is a reference unit for leukemia treatment of adults in the lubelskie voivodeship (region).

Exclusion criteria were as follows: patients under the age of 18 years old, patients receiving stem cell transplantation or radiotherapy or suffering from hematological diseases other than leukemia. Patients who started palliative health care or whom general state made dental examination impossible were not eligible for the study.

Initial and following dental examinations were performed by the same examiner—a dental practitioner, with 5 years of clinical experience. The examiner received appropriate training and was calibrated by an experienced clinician, a dentist too. Intraexaminer reproducibility was measured for this examiner and proved to be sufficient to perform the study. 

The study included 51 women and 51 men aged 22 to 72 (54.07 ± 10.33). The time that has elapsed since the patients was diagnosed was between 1 and 10 years with following diagnoses: Acute myelogenous leukemia (AML)—55 patients (53.92%), Chronic lymphocytic leukemia (CLL)—17 patients (16.67%), Acute lymphoblastic leukemia (ALL)—16 patients (15.69%), Chronic myelogenous leukemia (CML)—10 patients (9.80%), Acute promyelocytic leukemia (APL)—3 patients (2.94%), Chronic hairy cell leukemia (HCL)—1 patient (0.98%). The simplified division of leukemia into its chronic and the acute forms was used for statistical analysis. In 28 patients (27, 45%), chronic leukemia was stated; acute leukemia was diagnosed in 74 patients (72, 55%). All clinical data of patients, including laboratory test results, information about the applied treatment and its course, were obtained from hospital records. All participants provided written informed consent prior to enrollment in the study.

### 2.2. Dentition State Assessment

The initial examination, in which dentition state was assessed, was carried out before the next phase of leukemia treatment, e.g., before the next cycle of chemotherapy, right after medical qualification for the chemotherapy cycle. The following study was conducted after the chemotherapy cycle had been completed (after 7–14 days, depending on type of leukemia and the treatment protocol).

Examination took place at the hospital conditions, utilizing head torch and compressed air can. A basic dental diagnostic kit and a WHO 621 probe was used. Dental index (DMFT), commonly used in the clinical diagnostics, was calculated and used in accordance with WHO criteria [14] to evaluate the state of dentition. The DMFT index comprised decayed (D), missing (M), and filled or crowned (F) teeth (T) and added them to a final sum (possible range 0–32). A decayed tooth (D) and cavitated caries lesions were recorded when the tooth surface had an unmistakable cavity, detectably softened wall or undermined enamel (including secondary caries and temporary fillings). Initial caries (white spot lesions) were not considered in the present study. Restorations were recorded as “F” and missing teeth as “M” only when applied to caries-related reasons. Other enamel defects like fluorosis, amylogenesis imperfecta were not registered. All procedures performed in the study were in accordance with the ethical standards of the institutional and national research committee and with the 1964 Helsinki declaration and its later amendments or comparable ethical standards.

### 2.3. Statistical Analysis

The statistical analysis was carried out with the Statistica 12 software package (StatSoft, Tulsa, OK, USA). The calculation for quantitative traits included range of values (min., max), arithmetic mean (M), and standard deviation (SD). Differences between the compared groups for quantitative traits were verified with tests (depending on the stated distribution): for dependent variables—Students’-t, Wilcoxon, for independent variables—Student’s-t, Mann–Whitney U test. The differences in the prevalence of the analyzed traits between particular groups were tested with χ^2^ test (Fisher’s χ^2^) test). The study adopted a 5% risk of error; therefore, statistically significant differences were those of *p* < 0.05.

## 3. Results

### 3.1. Chemotherapy Cycle Versus DMFT Index

The mean DMFT value in leukemic patients before and after hematological treatment was 24.00 ± 6.17. There was no statistically significant change in the DMFT index after chemotherapy cycle. Dental caries incidence expressed as D number equaled 0 to 17 both at the first and the second appointment. The mean D index in the first examination was 4.48 ± 4.65, whereas after the treatment it was 4.06 ± 4.49. The reduction in D value after hematological treatment was statistically significant. The index of missing teeth (M) was 16.01 ± 9.81 in the first examination and 16.26 ± 9.78 in the follow-up examination and the change in its value was statistically significant. The mean number of teeth with fillings (F) was 3.51 ± 4.55 and 3.68 ± 4.68 in the first and second examinations, respectively. The change in those numbers was statistically significant (Table 1).

### 3.2. Changes in Hematological Parameters after Chemotherapy in Relation to the State of the Dentition

Based on the difference between the values in hematological parameters after the chemotherapy cycle and the values before the treatment—patients were divided into two groups: 1. Patients with lower or unchanged hematological parameters (↓) after treatment; 2. Patients with higher hematological parameters (↑) after treatment.

DMFT index and its components (D, M, F) were analyzed depending on the direction of changes in hematological parameters (↓ vs. ↑).

#### 3.2.1. Platelets (PLT)

No statistically significant difference was found for DMFT index depending on the direction of change in the platelet count in patients after hematological treatment. Significantly higher values of the caries index (D) were found both in the first and in the second study in patients whose platelet count increased after chemotherapy (Table 2).

#### 3.2.2. White Blood Cells (WBC)

No statistically significant difference was found for DMFT index depending on the direction of change in the white blood cells count in patients both before and after chemotherapy cycle. Statistically significantly higher values of the caries index D (decayed) were observed in patients with white blood cells count increase both before and after chemotherapy (Table 3).

#### 3.2.3. Neutrophilic Granulocytes (Neu)

The difference in DMFT value was statistically significant depending on the direction of change of neutrophils in the studied patients, both before and after the chemotherapy. The change in the values of D (decayed) and M (missing) indices was also statistically significant (Table 4).

#### 3.2.4. Lymphocytes (Lym)

Both before and after chemotherapy, the change in the number of lymphocytes did not correlate with the significant change of DMFT index. On the other hand, a significantly higher D index was found in patients whose lymphocyte count increased after the treatment (Table 5).

#### 3.2.5. Monocytes (Mono)

The change in the number of monocytes correlated with a significant change of the values of indicator—both before and after treatment—DMFT, D, M (Table 6).

#### 3.2.6. Eosinophilic Granulocytes (Eos)

When assessing the change in the number of eosinophils, a significant difference was found in the DMFT value in patients after the applied hematological treatment. The mean DMFT value in people with decreased eosinophil counts was 24.60 ± 6.23, while in patients with an increase in eosinophils, the mean DMFT was 22.04 ± 5.66 (Table 7).

#### 3.2.7. Basophils (Bas)

In patients with an increased number of basophils, a significantly higher value of F (filled) was found before and after chemotherapy (Table 8).

#### 3.2.8. Red Blood Cells (RBC)

There was no significant difference in DMFT index between patient groups both before and after chemotherapy regardless of the direction of change in red blood cells (Table 9).

### 3.3. Type of Leukemia in Relation to Changes in Dentition

DMFT index was also assessed depending on the type of leukemia. In patients with chronic leukemia, a significantly higher value of F index (filled teeth) was found compared to patients with acute leukemia, both before and after chemotherapy (Table 10). 

### 3.4. Duration of the Disease and the Changes in Dentition State (up to the Median, Inclusive—3 Years vs. above the Median)

Depending on the duration of the disease, the values of DMFT index was assessed. A significant correlation between the disease duration was found on the DMFT index values before and after hematological treatment. The mean DMFT value was higher in people with longer duration of the disease and was 26.00 ± 5.78 (Table 11).

### 3.5. Patients’ Age and the Changes in Dentition State (Up to the Median Inclusive—56 Years vs. above the Median)

The correlation between patients’ age on DMFT values was assessed. A statistically significantly higher M number value was found before and after hematological treatment in older patients. However, a statistically significantly higher value of F number before and after chemotherapy was found in younger patients (Table 12).

### 3.6. Gender and the Condition of the Oral Cavity

There was no statistically significant difference in the values of DMFT index depending on the gender of patients, neither before nor after hematological treatment (Table 13).

## 4. Discussion

The studies examining the dentition in adult patients with leukemia are rare, and up until now, the data of the oral health situation has been insufficient. Oral manifestations and initial symptoms are often important in the early detection of leukemia; in approximately 25% of the patients with AML, dentists are involved in the diagnosis. Apart from this, poor oral health is associated with a higher incidence of systemic infections and complications in patients suffering from leukemia. Accordingly, appropriate dental therapy before chemotherapy or stem cell transplantation can decrease the rate of septicemia cases and its fatal consequences [7,15,16,17,18,19].

In interpreting results of the present study, the low number of available data has to be considered. While there are several publications on the oral health status of children suffering from leukemia [14,17,20,21,22], not many investigations are focused on adult patients. Recent papers primarily describe case studies or retrospective studies with focus on AML and ALL and are not able to illustrate the oral health situation of patients before and after chemotherapy in different types of leukemia [23,24,25,26].

Busjan et al. conducted their research on 39 patients newly diagnosed with acute leukemia (ALL and AML) and 38 control patients that were matched to the leukemic patients by age, gender and smoking habits. The dental examination of the study group took place a few days after they were diagnosed—before beginning any treatment of the disease. The researchers claimed that there was no significant difference in DMFT values between the leukemic (combined ALL and AML) patients and control group. (18.69 ± 6.38 vs. 16.62 ± 7.4). Moreover, no significant difference in DMFT scores was found between patients suffering from AML and ALL (19.54 ± 5.06 vs. 17.00 ± 8.43). In our study, patients with leukemia (all types) both before and after the chemotherapy cycle presented mean DMFT index value of 24.00 ± 6.17. Moreover, we did not find any significant difference between DMFT values of 74 patients with acute and 28 patients with chronic leukemia (24.03 ± 5.85 vs. 23.93 ± 7.07) neither before nor after the treatment. By analyzing individual components of the index, Busjan et al. found significantly higher D and M values in patients with leukemia than in control groups. The mean age of patients in both studies was similar (55.61 ± 17.01 vs. 54.07 ± 10.33 in our study) [25].

According to Meyer et al., who conducted a study on immunocompromised patients, no statistically significant differences were found in DMFT index scores between groups of patients with leukemia—not in AML or ALL (53 patients, with either AML or ALL, mean DMFT 18.8 ± 9.0), systemic lupus erythematosus, heart transplant recipients or the control group. Presented value of mean DMFT index was similar to the research conducted by Busjan et al. [26].

In our study, the mean M (missing teeth) value of leukemic patients was 16.01 ± 9.81 before and 16.26 ± 9.78 after chemotherapy cycle. Comparing this result with the general population of different countries in this age group (45–64 years), it can be seen that patients from the present study on average have more teeth missing than people from a similar age group without this diagnosis. Suffering from leukemia may be considered one of the reasons of this situation. For example, in 855 citizens of Georgia aged 45–64, mean M value was 6.53 ± 6.47 [27], and 1945 Hungarian citizens from the general population who participated in the study had M mean value of 9.07 ± 7.24 [28].

According to the latest data of the Polish Ministry of Health, mean M index value in age group 35–44 living in a city was 3.1 ± 4.0 in 2010 and 1.2 ± 2.0 in 2017. Mean M value for Lublin voivodeship in this age group was 10.4 ± 4.4 in 2010 and 3.7 ± 1.8 in 2017. For age group 65–74 living in a city, the value of M index was 21.4 ± 7.9 in 2009 and 13.9 ± 8.8 in 2019. Mean M value for Lublin voivodeship for 2009 is missing, while in 2019 it was 12.5 ± 7.2. Data for population aged 45–64 is lacking [29].

Consequently, knowledge of the oral conditions of patients is insufficient, and the need of treatment for diagnosed patients appears unclear. Data from the US National Cancer Institute claims that some cancer centers encourage tooth brushing and flossing, while others indicate the interruption of brushing and flossing when blood components have a drop below specified limits (e.g., platelets < 30,000 cells/mm^3^). However, according to the institute itself, there is no evidence in the literature regarding the best approach. The centers providing strategy argue that the benefits of proper brushing and proper flossing outweigh the risks because the interruption of routine oral hygiene increases the risk of infection, and this could promote bleeding as well as increase the risk of local and systemic infection [7,30]. There is an increased risk of severe infections due to immunosuppression accompanying underlying disease and its treatment in patients with leukemia. In this respect, bacteremia from various sources is an important problem. Diseases of the oral cavity, especially decayed teeth and periodontitis, are associated with the development of bacteria with high pathological potential. That factor allows the entry of a large number of bacteria into the blood circulation, even during daily routine procedures such as oral hygiene or chewing. It has been found that the high incidence of gingivitis or periodontitis is an independent risk factor for infectious complications during chemotherapy [31,32,33]. In addition, dental interventions, especially oral surgery during chemotherapy, can have serious consequences such as bleeding complications. Therefore, it is recommended to perform dental clearance before induction chemotherapy to avoid both infectious complications and the need for dental intervention during therapy [34]. This is also confirmed by the study of dental clinicians, in which dental rehabilitation has priority over induction therapy [35]. According to these recommendations, the oral mucosa, dentition, periodontium should be carefully examined before chemotherapy.

Diagnostic and therapeutic difficulties in the oral cavity are additionally caused by exacerbations and remissions of the general disease. Maintaining proper oral hygiene by the patient may be difficult due to pain, intense bleeding and ulcerations often occurring after chemotherapy. It is necessary to thoroughly cleanse the mouth and use rinses to inhibit the growth of micro-organisms to reduce the severity of the disease symptoms and side effects of therapy. Treatment should lead to a reduction in pain, dry mouth and promote healing of necrotic changes. So far, no universal way to prevent or treat inflammation of the periodontium has been found in patients undergoing chemotherapy. The use of rinses containing chlorhexidine in this group of patients did not bring the expected results. Clinical observations show that salivary substitutes, rinsing the mouth with sterile water, irrigations with sodium bicarbonate and the use of local anesthetics are effective [35,36,37,38].

In this study, statistically significant changes in D, M, F values resulted from restorative procedures (treatment of carious cavities) or tooth extractions. Therefore, awareness of the importance of dental treatment along with the medical management of these patients should be spread. A dental surgeon may be the first doctor to see a patient with hematopoietic and lymphatic disorders. It is very important to assess the changes in the mouth of a patient who is already undergoing treatment for a hematological disease [25,39,40,41].

The study is limited by the fact that dental examination was not performed at the dental office condition. Moreover, examined patients are people with poor general health and compromised dental hygiene. Population of patients being treated in this hospital consist generally of people from the voivodeship and cannot be extrapolated to the whole country, and only patients over 22 years of age have been considered.

However, the strength of present study is the sample size, which is relatively high for the studies focused on similar subject.

Further studies are necessary to fully understand the causes of tooth loss in leukemic patients and provide solutions to prevent it.

## 5. Conclusions

1. On the basis of the obtained values of dental indices (D, M, F, DMFT), it was observed that patients’ dentition state changed after the cycle of chemotherapy that was used. 

2. The change in hematological parameters (apart from RBC) after chemotherapy correlated with change in DMFT index and/or its components.

3. The high prevalence of dental caries confirms the need for early and consistent dental treatment of patients with leukemia, especially considering hematological therapy. 

4. Patients suffering from leukemia require permanent dental control. It can mitigate pathological processes in the oral cavity related with the disease and its treatment. Such management will allow the prevention of local complications such as tooth loss and will also affect patients’ general state.

## Figures and Tables

**Table 1:** DMFT index and its components values.

		n	min	max	M	SD	Me	*p*
DMFT	Before treatment	102	10.00	32.00	24.00	6.17	24.00	1.000
	After treatment	102	10.00	32.00	24.00	6.17	24.00	
D	Before treatment	102	0.00	17.00	4.48	4.65	3.00	<0.001
	After treatment	102	0.00	17.00	4.06	4.49	2.50	
M	Before treatment	102	0.00	32.00	16.01	9.81	13.50	<0.001
	After treatment	102	0.00	32.00	16.26	9.78	14.00	
F	Before treatment	102	0.00	15.00	3.51	4.55	1.00	<0.01
	After treatment	102	0.00	15.00	3.68	4.68	1.50	

**Table 2:** Platelet count change and DMFT index before (1) and after (2) chemotherapy.

	↓-PLT			↑-PLT			*p*
	*n*	M	SD	*n*	M	SD	
DMFT (1)	75	24.35	6.19	27	23.04	6.15	0.3204
D (1)	75	3.76	4.60	27	6.48	4.26	0.0042
M (1)	75	17.17	9.80	27	12.78	9.25	0.0772
F (1)	75	3.41	4.35	27	3.78	5.15	0.9456
DMFT (2)	75	24.35	6.19	27	23.04	6.15	0.3204
D (2)	75	3.36	4.37	27	6.00	4.31	0.0039
M (2)	75	17.44	9.72	27	13.00	9.35	0.0687
F (2)	75	3.55	4.44	27	4.04	5.37	0.9365

**Table 3:** White blood cells count change and DMFT index before (1) and after (2) chemotherapy.

	↓-WBC			↑-WBC			*p*
	*n*	M	SD	*n*	M	SD	
DMFT (1)	60	24.90	6.03	42	22.71	6.22	0.2184
D (1)	60	2.92	3.39	42	6.71	5.30	0.0001
M (1)	60	17.55	10.30	42	13.81	8.71	0.1116
F (1)	60	4.43	5.43	42	2.19	2.39	0.1428
DMFT (2)	60	24.90	6.03	42	22.71	6.22	0.2184
D (2)	60	2.55	3.16	42	6.21	5.21	0.0002
M (2)	60	17.75	10.26	42	14.14	8.73	0.1139
F (2)	60	4.60	5.55	42	2.36	2.56	0.1553

**Table 4:** Neutrophilic granulocytes count change and DMFT index before (1) and after (2) chemotherapy.

	↓-Neu			↑-Neu			*p*
	*n*	M	SD	*n*	M	SD	
DMFT (1)	66	25.20	6.14	36	21.81	5.68	0.0330
D (1)	66	3.47	3.52	36	6.33	5.82	0.0222
M (1)	66	17.79	10.21	36	12.75	8.20	0.0319
F (1)	66	3.94	5.19	36	2.72	2.96	0.5037
DMFT (2)	66	25.20	6.14	36	21.81	5.68	0.0330
D (2)	66	3.05	3.34	36	5.92	5.65	0.0179
M (2)	66	18.02	10.22	36	13.06	8.11	0.0339
F (2)	66	4.14	5.33	36	2.83	3.07	0.5240

**Table 5:** Lymphocytes count change and DMFT index before (1) and after (2) chemotherapy.

	↓-Lym			↑-Lym			*p*
	*n*	M	SD	*n*	M	SD	
DMFT (1)	65	24.91	5.98	37	22.41	6.26	0.1410
D (1)	65	3.51	3.93	37	6.19	5.33	0.0069
M (1)	65	17.57	9.60	37	13.27	9.70	0.0547
F (1)	65	3.83	4.90	37	2.95	3.87	0.6286
DMFT (2)	65	24.91	5.98	37	22.41	6.26	0.1410
D (2)	65	3.14	3.69	37	5.68	5.30	0.0097
M (2)	65	17.78	9.53	37	13.59	9.76	0.0552
F (2)	65	3.98	5.01	37	3.14	4.05	0.6839

**Table 6:** Monocytes count change and DMFT index before (1) and after (2) chemotherapy.

	↓-Mono			↑-Mono			*p*
	*n*	M	SD	*n*	M	SD	
DMFT (1)	71	25.25	5.74	31	21.13	6.26	0.0058
D (1)	71	3.56	3.91	31	6.58	5.53	0.0049
M (1)	71	17.87	10.31	31	11.74	6.98	0.0085
F (1)	71	3.82	5.17	31	2.81	2.60	0.7242
DMFT (2)	71	25.25	5.74	31	21.13	6.26	0.0058
D (2)	71	3.18	3.64	31	6.06	5.57	0.0142
M (2)	71	18.10	10.29	31	12.06	6.98	0.0084
F (2)	71	3.97	5.31	31	3.00	2.71	0.7297

**Table 7:** Eosinophilic granulocytes count change and DMFT index before (1) and after (2) chemotherapy.

	↓-Eos			↑-Eos			*p*
	*n*	M	SD	*n*	M	SD	
DMFT (1)	78	24.60	6.23	24	22.04	5.66	0.0307
D (1)	78	4.54	4.79	24	4.29	4.25	0.6586
M (1)	78	16.68	10.11	24	13.83	8.60	0.3706
F (1)	78	3.38	4.47	24	3.92	4.91	0.6730
DMFT (2)	78	24.60	6.23	24	22.04	5.66	0.0307
D (2)	78	4.14	4.60	24	3.79	4.20	0.8406
M (2)	78	16.91	10.03	24	14.17	8.78	0.3581
F (2)	78	3.55	4.54	24	4.08	5.19	0.6788

**Table 8:** Basophils count change and DMFT index before (1) and after (2) chemotherapy.

	↓-Bas			↑-Bas			*p*
	*n*	M	SD	*n*	M	SD	
DMFT (1)	63	23.98	6.30	39	24.03	6.05	0.9588
D (1)	63	4.05	4.39	39	5.18	5.03	0.2766
M (1)	63	15.73	9.68	39	16.46	10.12	0.6128
F (1)	63	4.21	4.81	39	2.38	3.92	0.0198
DMFT (2)	63	23.98	6.30	39	24.03	6.05	0.9588
D (2)	63	3.68	4.27	39	4.67	4.82	0.3490
M (2)	63	15.92	9.75	39	16.82	9.94	0.4911
F (2)	63	4.38	4.92	39	2.54	4.06	0.0152

**Table 9:** Red blood cells count change and DMFT index before (1) and after (2) chemotherapy.

	↓-RBC			↑-RBC			*p*
	*n*	M	SD	*n*	M	SD	
DMFT (1)	76	23.55	6.44	26	25.31	5.21	0.2542
D (1)	76	4.50	4.72	26	4.42	4.52	0.8268
M (1)	76	15.21	10.29	26	18.35	7.96	0.0868
F (1)	76	3.84	4.58	26	2.54	4.44	0.1313
DMFT (2)	76	23.55	6.44	26	25.31	5.21	0.2542
D (2)	76	4.12	4.59	26	3.88	4.26	0.9816
M (2)	76	15.42	10.24	26	18.73	7.95	0.0748
F (2)	76	4.01	4.71	26	2.69	4.52	0.1717

**Table 10:** Leukemia type and DMFT index before (1) and after (2) chemotherapy.

	Chronic			Acute			*p*
	*n*	M	SD	*n*	M	SD	
DMFT (1)	28	23.93	7.07	74	24.03	5.85	0.7901
D (1)	28	4.32	3.63	74	4.54	5.00	0.8631
M (1)	28	15.11	7.56	74	16.35	10.56	0.6800
F (1)	28	4.50	4.32	74	3.14	4.61	0.0077
DMFT (2)	28	23.93	7.07	74	24.03	5.85	0.7901
D (2)	28	3.96	3.69	74	4.09	4.78	0.7872
M (2)	28	15.36	7.79	74	16.61	10.46	0.6023
F (2)	28	4.61	4.37	74	3.32	4.77	0.0195

**Table 11:** Duration of the disease and DMFT index before (1) and after (2) chemotherapy.

	≤3			>3			*p*
	*n*	M	SD	*n*	M	SD	
DMFT (1)	63	22.76	6.13	39	26.00	5.78	0.0189
D (1)	63	4.63	5.01	39	4.23	4.06	0.7698
M (1)	63	15.05	9.92	39	17.56	9.55	0.1931
F (1)	63	3.08	4.32	39	4.21	4.89	0.0734
DMFT (2)	63	22.76	6.13	39	26.00	5.78	0.0189
D (2)	63	4.13	4.77	39	3.95	4.06	0.9890
M (2)	63	15.33	9.84	39	17.77	9.61	0.2295
F (2)	63	3.30	4.52	39	4.28	4.93	0.1369

**Table 12:** Patients’ age and DMFT index before (1) and after (2) chemotherapy.

	≤56			>56			*p*
	*n*	M	SD	*n*	M	SD	
DMFT (1)	54	23.07	6.07	48	25.04	6.18	0.0773
D (1)	54	5.06	4.63	48	3.83	4.64	0.1062
M (1)	54	13.65	9.19	48	18.67	9.90	0.0103
F (1)	54	4.37	4.52	48	2.54	4.44	0.0037
DMFT (2)	54	23.07	6.07	48	25.04	6.18	0.0773
D (2)	54	4.65	4.56	48	3.40	4.36	0.1128
M (2)	54	13.89	9.23	48	18.94	9.78	0.0093
F (2)	54	4.54	4.66	48	2.71	4.56	0.0063

**Table 13:** Patients’ gender and DMFT index before (1) and after (2) chemotherapy.

	Female			Male			*p*
	*n*	M	SD	*n*	M	SD	
DMFT (1)	51	24.63	5.70	51	23.37	6.61	0.6782
D (1)	51	3.65	3.78	51	5.31	5.29	0.3770
M (1)	51	17.14	8.74	51	14.88	10.74	0.1569
F (1)	51	3.84	4.83	51	3.18	4.29	0.4142
DMFT (2)	51	24.63	5.70	51	23.37	6.61	0.6782
D (2)	51	3.29	3.76	51	4.82	5.04	0.3236
M (2)	51	17.31	8.84	51	15.22	10.63	0.2059
F (2)	51	4.02	5.00	51	3.33	4.36	0.4297

## Data Availability

Not applicable.

## References

[1] Lupi S.M., Rodriguez Y., Baena A., Cervino G., Todaro C., Rizzo S. (2018). Long-Term effects of acute Myeloid Leukemia Treatment on the Oral System in Pediatric Patient. Open Dent. J..

[2] Zimmermann C., Meurer M.I., Grando L.J., Gonzaga Del Moral J.Â., da Silva Rath I.B., Schaefer Tavares S. (2015). Dental treatment in patients with leukemia. J. Oncol..

[3] Belver L., Ferrando A. (2016). The genetics and mechanisms of T cell acute lymphoblastic leukaemia. Nat. Rev. Cancer.

[4] Padmin I.C., Bai K.Y. (2014). Oral and Dental Considerations in Pediatric Leukemic Patient. ISRN Hematol..

[5] Brazelton J., Louis P., Sullivan J., Peker D. (2014). Temporomandibular joint arthritis as an initial presentation of acute myeloid leukemia with myelodysplasia-related changes: A report of an unusual case. J. Oral. Maxillofac. Surg..

[6] Ceccarelli G., Presta R., Benedetti L., Cusella De Angelis M.G., Lupi S.M., Rodriguez Y., Baena R. (2017). Emerging perspectives in scaffold for tissue engineering in oral surgery. Stem. Cells Int..

[7] Ptasiewicz M., Pawlowicz A.K., Tymczyna-Borowicz B. (2020). Chemotherapy and oral health in leukemic patients. Pol. J. Environ. Stud..

[8] Chen Y., Zhang J., Ma Q., Sun C., Ha S., Zhang F. (2016). Human health risk assessment and source diagnosis of polycyclic aromatic hydrocarbons (PAHs) in the corn and agricultural soils along main roadside in Changchun, China. Hum. Ecol. Risk Assess..

[9] Little J.W., Falace D.A., Miller S.C., Rdodus N.L. (2008). Antibiotic prophylaxis in dentistry: An update. Gen. Dent..

[10] Sonis S.T. (2004). The pathobiology of mucositis. Nat. Rev. Cancer.

[11] Napenas J.J., Brennan M.T., Bahrani-Mougot F.K., Fox P.C., Lockhart P.B. (2007). Relationship between mucositis and changes in microflora during cancer chemotherapy. Oral Surg. Oral Med. Oral Pathol. Oral Radiol. Endod..

[12] Francisconi C.F., Caldas R.J., Oliveira Martins L.J., Fischer Rubira C.M., Da Silva Santos P.S. (2016). Leukemic oral manifestations and their management. Asian Pac. J. Cancer Prev..

[13] Hochhaus A., Erben P., Ernst T., Mueller M.C. (2007). Resistance to targeted therapy in chronic myelogenous leukemia. Semin. Hematol..

[14] WHO (1997). World Health Organization: Oral Health Surveys, Basic Methods.

[15] Khaled S., Malki M., Marcucci G. (2016). Acute myeloid leukemia: Biologic, prognostic, and therapeutic insights. Oncology (Williston Park).

[16] De Felice F., Grapulin L., Musid D., Pomponi J., Di Felice C., Iori A.P., Bertaina A., Tombolini V. (2016). Treatment complications and long-term outcomes of total body irradiation in patients with acute lymphoblastic leukemia: A single institute experience. Anticancer Res..

[17] Mathur V.P., Dhillon J.K., Kalra G., Athur V.P. Oral health in children with leukemia. Indian J. Palliat. Care.

[18] Parisi E., Drazinin J., Stoopler E., Schuster S.J., Poster D., Scollecito T.P. (2002). Acute myelogenous leukemia: Advances and limitation of treatment. Oral Surg. Oral Med. Oral Pathol..

[19] Ravikumar R., Manohar R., Latha S.M., Scott J.X. (2016). Gingival hypertrophy in a child: Expect the unexpected. Indian J. Dent..

[20] Valéra M.-C., Noirrit-Esclassan E., Pasquet M., Vaysse F. (2014). Oral complications and dental care in children with acute lymphoblastic leukaemia. J. Oral Pathol. Med..

[21] Javed F., Utreja A., Bello Correa F.O., Al-Askar M., Hudieb M., Qayyum F., Al-Rasheed A., Almas K., Al-Hezaimi K. (2012). Oral health status in children with acute lymphoblastic leukemia. Crit. Rev. Oncol. Hematol..

[22] Hegde A.M., Joshi S., Rai K., Shetty S. (2011). Evaluation of oral hygiene status, salivary characteristics and dental caries experience in acute lymphoblastic leukemic (ALL) children. J. Clin. Pediatr. Dent..

[23] Angst P.D.M., Dutra D.A.M., Moreira C.H.C., Kantorski K.Z. (2012). Periodontal status and its correlation with haematological parameters in patients with leukaemia. J. Clin. Periodontol..

[24] Gazi M., Ashri N., Lambourne A. (1991). Oral health care of Saudi leukemic patients. Ann. Saudi. Med..

[25] Busjan R., Hasenkamp J., Schmalz G., Haak R., Trümper L., Ziebolz D. (2018). Oral health status in adult patients with newly diagnosed acute leukemia. Clin. Oral Investig..

[26] Meyer U., Kleinheinz J., Handschel J., Kruse-Lösler B., Weingart D., Joos U. (2000). Oral findings in three different groups of immunocompromised patients. J. Oral Pathol. Med..

[27] Tsitaishvili L. (2014). Assessment of Caries Prevalence and Related Risk Factors Among the Adult Population of Georgia by Age and Gender. Dent. Med. Probl..

[28] Madléna M., Hermann P., Jáhn M., Fejérdy P. (2008). Caries prevalence and tooth loss in Hungarian adult population: Results of a national survey. BMC Public Health.

[29] Olczak-Kowalczyk D. (2021). Choroba Próchnicowa i Stan Tkanek Przyzębia Populacji Polskiej. Podsumowanie Wyników Badań z Lat 2016–2019.

[30] Dabaja B.S., Specht L., Yahalom J. (2018). Lymphoblastic Lymphoma: Guidelines from the International Lymphoma Radiation Oncology Group (ILROG). Int. J. Radiat. Oncol Biol. Phys..

[31] Harris M.H., Czuchlewski D.R., Arber D.A., Czader M. (2019). Genetic Testing in the Diagnosis and Biology of Acute Leukemia. Am. J. Clin. Pathol..

[32] Babu S.P., Kashyap V., Sivaranjani P., Agila S. (2001). An undiagnosed case of acute myeloid leukemia. J. Indian Soc. Oncol..

[33] Epstein J.B., Stevenson-Moore P. (2001). Periodontal disease and periodontal management in patients with cancer. Oral Oncol..

[34] Chałas R., Wójcik-Chęcińska I., Woźniak M.J., Grzonka J., Święszkowski W., Kurzydłowski K.J. (2015). Dental plaque as a biofilm—A risk in oral cavity and methods to prevent. Postepy Hig. Med. Dosw..

[35] Potting C.M., Uitterhoeve R., Opreimer W.S., Van Achterberg T. (2006). The effectiveness of commonly used mouthwashes for the prevention of chemotherapy−induced oral mucositis: A systemic review. Eur. J. Cancer Care.

[36] Subramanian P., Baku K.L., Nagarathana J. (2008). Oral manifestations in acute lymphoblastic children under chemotherapy. J. Clin. Pediatr. Dent..

[37] Velten D.B., Zandonade E., Barros M.H.M. (2017). Prevalence of oral manifestations in children and adolescents with cancer submitted to chemotherapy. BMC Oral Health.

[38] Allareddy V., Prakasam S., Allareddy V., Martinez-Schlurmann N.I., Rampa S., Nalliah R.P., Eswaran S.V., Elangovan S. (2015). Poor oral health linked with increased risk of infectious complications in adults with leukemia. J. Mass. Dent. Soc..

[39] Heesch S., Neumann M., Schwattz S. (2013). Acute leukemias of ambiguous lineage in adults: Molecular and clinical charakterization. Ann. Hematol..

[40] Carrega G., Castagnola E., Canessa A., Argenta P., Haupt R., Dini G., Garaventa A. (1994). Herpes simplex virus and oral mucositis in children with cancer. Support Care Cancer.

[41] Doumbia M., Uwingabiye J., Bissan A., Rachid R., Benkirane S., Masrar A. (2016). Epidemiological, clinical, cytologic and immune phenotypic aspects of acute leukemia in children: The experience at the hematology laboratory of IBN SINA University Hospital Center. Pan Afr. Med. J..

